# Genome Sequence of *Talaromyces atroroseus*, Which Produces Red Colorants for the Food Industry

**DOI:** 10.1128/genomeA.01736-16

**Published:** 2017-03-02

**Authors:** Ulf Thrane, Kasper Bøwig Rasmussen, Bent Petersen, Simon Rasmussen, Thomas Sicheritz-Pontén, Uffe Hasbro Mortensen

**Affiliations:** a Department of Biotechnology and Biomedicine, Technical University of Denmark, Kongens Lyngby, Denmark; b Department of Bio and Health Informatics, Technical University of Denmark, Kongens Lyngby, Denmark

## Abstract

*Talaromyces atroroseus* is a known producer of *Monascus* colorants suitable for the food industry. Furthermore, genetic tools have been established that facilitate elucidation and engineering of its biosynthetic pathways. Here, we report the draft genome of a potential fungal cell factory, *T. atroroseus* IBT 11181 (CBS 123796).

## GENOME ANNOUNCEMENT

The genus *Talaromyces* primarily contains saprophytic fungi and encompasses medically and industrially relevant species such as the opportunistic human pathogen *T. marneffei* (formerly *Penicillium marneffei*), species with high production of cellulolytic enzymes, i.e., *T. cellulolyticus* (1), as well as the interesting pigment-producing species *T. atroroseus* (2). Several strains of *T. atroroseus* and closely related species are recognized as potential cell factories for *Monascus* pigment production, as they may serve as mycotoxin-free alternatives to *Monascus* spp. (2–4). 

*T. atroroseus* IBT 11181 was originally isolated from red sweet bell pepper bought in a Danish supermarket and is deposited in the CBS collection at CBS-KNAW, Utrecht, the Netherlands, as CBS 123796 and CBS 238.95. We intend to implement this isolate as a model for *T. atroroseus* by investigating its growth physiology (5), by establishing genetic tools (6), and by reporting here the full-genome sequence of *T. atroroseus* IBT 11181.

Genomic DNA was extracted from the mycelium with a slightly modified protocol of the cetyltrimethylammonium bromide method used by Fulton et al. (7). The *T. atroroseus* IBT 11181 genome was sequenced using an Illumina HiSeq 2000 platform on a 180-bp paired-end library and a 6-kb mate-paired library both with reads of 2 × 100 bp by Beijing Genome Institute (BGI), Hong Kong. Sequencing depth was 193×, and assembly of the genome was performed with the ALLPATHS-LG algorithm (8). The final assembly resulted in 48 scaffolds with a G+C content of 44.35% and a total assembly size of 30.85 Mb corresponding to 93% of the estimated genome size from *k*-mer spectral analysis. The minimum number of sequences making up 50% of the genome assembly was seven, and the *N*_50_ length was 1,577,401 bp. The CEGMA pipeline (9) identified 242 of the 248 core eukaryotic genes, assessing the genome assembly completeness to be 97.58%. This indicated that the draft genome assembly was good with a high completeness and was valid to use for whole-genome analysis.

Gene-calling of the genome was performed using a pipeline of first masking the genome with RepeatMasker version 4.0.5 (Institute for Systems Biology, Seattle, WA, USA; http://www.repeatmasker.org), and then gene-calling with AUGUSTUS version 3.0.3 (10, 11), FGENESH version 3.1.2 (SoftBerry) (12), and GeneMark-ES (13). The individual *ab initio* gene predictions were merged into a consensus gene prediction using EVidenceModeler (14), resulting in a total of 9,519 protein-encoding genes serving as the final gene prediction. The genome sequence reported here represents a useful resource for further research into the metabolism of *T. atroroseus* and its potential as a cell factory for colorant production.

### Accession number(s).

This whole-genome shotgun project has been deposited at DDBJ/ENA/GenBank under the accession number LFMY00000000. The version described in this paper is the first version, LFMY01000000.
